# Interesting Cases of Apoplexy, with Dissections

**Published:** 1830-01-01

**Authors:** 


XXVIII.

### INTERESTING CASES OF APOPLEXY WITH DISSECTIONS.*

Of late years, the diseases of the brain have attracted great attention, and in truth they deserve it. Much has been done in morbid anatomy but much remains to do, and we know not a more promising field than the diseases of the brain and the circulating system, especially the connexion that exists between them. We have been turning our attention to this point of late, and perhaps some day we may lay the result of our observations before the profession. The following cases recorded by Dr. Mills are not unworthy of notice, as they illustrate some points connected with that formidable and too frequent disease—apoplexy.

*Case* 1. Mr. L——, æt. 40, of sedentary habits, complains (June 6th, 1817) of pain, heat, and heaviness in the head—weakness of the muscles of right eye-lid and right side of mouth—indistinct speech—indigestion—pulse 68, irregular—skin cool—tongue yellowish—fæces dark coloured. Has suffered from these symptoms for the last four months, has been attacked with epileptic fits within the last six weeks, and

* Mills on the Morbid Appearances and Disorders of the Brain.

has at times been incoherent. *Leeches and cathartics.* Next day there was some stupor and occasional vomiting. *Blister to the nucha.* On the 8th there was paralysis of the right side of the face, and on the ninth delirium, which symptoms continued with little alteration till the 13th, when the ideas were less confused and the patient more disposed to conversation. On the 15th there came on strabismus, and on the following day coma, but on the 19th the patient was so far revived as to talk of going into the country. On the 20th, however, he was attacked with apoplexy and died on the morning of the 21st.

"*Dissection by Mr. M'Namara.* The mezentery is loaded with fat, and the bladder distended with urine.—The spleen is softer than natural, its peritoneal membrane bears evident marks of previous inflammation.—Liver, of a natural appearance.—Gall-bladder, distended with greenish yellow bile.—Kidneys, covered with fat and internally the calices are surrounded with fat.—Lungs and heart, of a natural appearance.—Pericardium contains half an ounce of a watery fluid.—The surface of the brain is highly vascular.—The lateral ventricles are considerably enlarged, and in them, at the base of the brain, and in the theca spinalis, are found three ounces of a serous fluid.—Plexus choroides pale."

It will be observed that in the foregoing case there was no hemiplegia, and no sanguineous partial effusion. Apoplexy from serous effusion into the ventricles, or even from mere sanguineous congestion of the venous system in the head is frequently unattended with hemiplegia, and rather appears to evince a disposition to give rise to coma and general paralysis. This is, cæteris paribus, a fact of considerable service in guiding our diagnosis, and even our practice with respect to blood-letting. In the following instances of hemiplegia there were sanguineous extravasations on the opposite side of the brain.

*Case 2.* Mr. B. æt. 55, a country gentleman, complains (Aug. 9, 1819) of head-ache, vertigo, heaviness about the head, accompanied by fever, and occasionally by pains in the stomach, nausea, or vomiting—face florid and flushed—heat of forehead great—pulse 96 and strong—palpitations at the heart—tongue yellowish and foul—fæces often morbid in appearance—urine frequently high coloured and depositing a pink or lateritious sediment. This gentleman has been accustomed to the use of fermented liquors; his complaint has been considered gouty, and various tonics, cordials, and bitters have been prescribed for it. *V S. ad* ℥xij.—*cathartics.* The head was relieved by the bleeding, and on the 10th twelve leeches were applied to the temples, with so much relief that he became anxious to return into the country. On the 13th, however, after riding on horseback, he became gradually attacked with hemiplegia of the left side, for which he was bled, leeched, and purged. Delirium and vomiting succeeded, then stupor, stertorous breathing, and death on the 17th.

"*Dissection by Mr. M'Namara.* The dura mater exhibits evident marks of excessive vascularity both arterial and venous.—The veins on the surface of the cerebrum are very turgid.—The convolutions of the cerebrum are remarkably depressed.—On making a section of the brain a preternatural number of red vessels are discovered. In the centre of the posterior lobe of the right hemisphere is found a cavity of the size of a hen's egg filled with coagulated blood. The walls of this cavity are formed of the substance of the cerebrum, which is softened and of a bright red-colour.—The blood appears to have been furnished from a number of minute vessels observed on its surface, and not from the rupture of any large vessel, similar to what is noticed on the surface of the intestines in melæna.—The edge of the plexus choroides has an hydatid-like appearance.—In the ventricles are about three drams of a serous fluid, and about six at the base of the cranium and in the theca vertebralis.—The cerebellum is preternaturally vascular.—The lungs are of a natural appearance.

"The heart is enlarged and softer than natural, and its right auricle and ventricle are filled with a dark-coloured fluid blood.

"The liver, when cut into, is found to be exceedingly vascular.—The gall-bladder contains two gall-stones, irregular in their shape and of the size of French beans; there are several others considerably smaller.—The spleen is enlarged, softened and gorged with dark blood.—A large portion of the mucous coat of the stomach near the cardia is preternaturally vascular, some of the vessels have a florid appearance."

As Dr. Mills observes, the stimulants and cordials exhibited for gout most probably sent this poor gentleman to ORCUS before his time. Had more judicious antiphlogistic regimen been pursued at first, life might have been prolonged.

*Case* 3. "A. H. a country-woman, had a fit of apoplexy.—She was naturally robust and sanguine, but for some months had an earthy unnatural complexion, was dispirited and complained of sick head-ach.—Four years before this seizure she had a violent inflammatory fever, with pain in the epigastrium, which yielded to copious bleeding and the antiphlogistic regimen.—It was succeeded by symptoms allied to mania. Though free from fever she talked incessantly, and never slept, but wandered about all night. Her manner was forward and immodest, and the powers of her mind were powerfully excited. She was prompt in her replies, positive in all her opinions, and on all subjects employed a copious eloquence. These symptoms yielded to free purging.

"After the attack she was insensible, and the right side was motionless, but she frequently lifted the left hand to her head, and the leg of the same side was violently moved. The power of swallowing was gone, but the sphincters were closed.—Free bleeding from the external jugular had no effect, and she survived the attack only twenty-four hours.

"DISSECTION. — The dura mater adhered firmly to the cranium.—The finer membranes were manifestly inflamed; between them there was an extensive effusion of bloody lymph, which reddened the surface of the cerebrum.

"As was suspected, the greatest mischief was on the left side of the brain; the anterior half of which was completely injected with blood.—A large coagulum, which had evidently proceeded from the rupture of innumerable diseased vessels, filled the left ventricle, and had broken down the thalamus nervi optici of that side, and the corpus striatum.

"There was serum in all the other ventricles.—The cerebellum was surrounded by water; its surface was deeply inflamed, and in its substance there were many apoplectic cells."

*Case* 4. Mr. F. æt. 72, full and plethoric, of sedentary habits, has been subject (date 1818) for years to indigestion, vertigo, acute head-aches, and occasional slight paralytic attacks of the fingers or arms. On the 13th of April he was seized with apoplexy followed by hemiplegia of the right side, and in a fortnight afterwards he died.

*Dissection by Mr. M'Namara*:——

"The dura mater is extremely vascular, and the veins on the surface of the cerebrum are very turgid; the Arachnoid membrane is raised from the pia mater by a serous effusion. — The substance of the brain is firmer than natural.—A considerable number of red vessels is discovered on making a section of the cerebrum.—The ventricles are enlarged and distended with a watery fluid.—In the middle lobe of the left hemisphere there is a regularly formed cavity, large enough to contain a hen's egg, this cavity is filled with grumous blood and its surface is of a bright claret colour.—On cutting through the cerebellum a small clot of blood is discovered.—About four ounces of a serous fluid are found in the ventricles, the base of the cranium and the theca spinalis.

"The integuments of the skull, chest and abdomen are œdematous. — The omentum is fatty.—In the lining membrane of the stomach and intestines are several red patches.—The bladder is thickened and diminished in size, and on its mucous coat are numerous red spots.—Lungs, healthy.—Heart, preternaturally large, a portion of the left mitral valve is thickened and contracted, and the semilunar valve of the aorta is thickened."

The foregoing cases though brief are extremely interesting; the following is one of a different character in some, though not all respects.

"J. L———, æt. 30, weak in intellect and of a spare habit, was for several years employed as an attorney's clerk, and, for some time, was involved in pecuniary difficulties. — His appetite was good but his bowels were constipated, and he was subject to head-aches.

"On the night of the twenty-first instant he was attacked with what he called the night-mare, which he ascribed to drinking cold water at bedtime.—At ten o'clock, a. m., on the twenty-second, he was seized with general convulsions, followed by stupor, stertorous breathing and dilatation of the pupils; spirit of turpentine was administered per os et anum, which acted as a cathartic.—About five o'clock p. m., the convulsions returned with violence; the wrists and ankles were considerably distorted, and the body was bent back with an inclination towards the right side; the vessels of the head and neck were turgid; the pupils were dilated, the tunica conjunctiva was red, and the breathing stertorous; there was frequent moaning and profuse general perspiration.—Thirty ounces of blood were taken from the jugular vein and temporal artery at nine o'clock, p. m.—The head was shaved and blistered, and a fetid injection administered.—The pulse became small and irregular, and though the turgescence of the vessels of the head subsided, yet the convulsions, stertor, moaning, and dilatation of the pupils continued.

"Death took place at eight o'clock on the morning of the twenty-third of July, about twenty-four hours after the first attack of convulsions.—At three o'clock, p. m., the same day, the body was examined by Dr. Peebles.

"The contents of the abdomen showed no marks of disease.

"The superior and lateral portions of the brain were of a dark red-colour; there were about two ounces of coagulated blood at the medulla oblongata, and a quantity of turbid serum flowed from the ventricles and from beneath the Arachnoid membrane.—The vessels of the pia mater were turgid. — The walls of the ventricles were dotted with numerous red points. — On removing the coagula from the under surface of the brain, the basilary artery was found ulcerated and ruptured, so as to admit a small quill into its canal below and behind the origin of the posterior cerebral arteries, the margin of the orifice was thick, and of a dull yellow-colour. — The diseased structure did not surround the whole artery, nor was there any appearance of an aneurismal sac.— About half an ounce of serum tinged with blood flowed from the spinal canal."

It is to be regretted that the account of the dissection is imperfect, the state of the blood-vessels of the brain, of the heart, and indeed of the circulating system generally not being specified. As it is, the case shews the description of symptoms usually produced by effusion of blood about the medulla oblongata, namely, convulsions rather than hemiplegia. With this we must conclude for the present.

